# A Note on Scottish Hospital History

**Published:** 1921-07-23

**Authors:** 


### A Note on Scottish Hospital History.

The supporters of Hospital Saturday in Leith are accustomed to attend an annual service in South Leith Parish Church, where they and representatives of Leith Hospital meet to give thanks and listen to an address. This year the "annual commemoration lecture" was delivered by the Rev. William Swan, B.D., who traced the history of the Leith hospitals from the time of King James VI. Beside St. Mary's Church there stood in the Middle Ages the monastery of St. Anthony, founded in 1430. Part of its revenue came from the duties on wine which entered Leith harbour. It was the only monastery of the Order in Scotland, and the monks took charge of the sick in memory of their patron, who had been credited with the cure of erysipelas, then called St. Anthony's fire. In 1592 the monastery was closed, and King James eventually presented the residue of its possessions to the minister and Kirk Session of South Leith to form King James VI. Hospital, which endured till 1830, when the building was demolished and the funds used to help needy widows. The Humane Society was founded in 1786, and the Destitute Sick Society, a little later, looked after the sick in their own homes. From a dispensary founded at the same period Leith Hospital seemed to have grown. In Quality Street a casualty and a fever hospital were once combined till 1846. £1,000 was left by John Stewart to build a fever hospital. In 1850 Leith Hospital was built, and added to in 1866, 1872, and 1889. The speaker also referred, in passing, to Trinity Hospital, Seafield Hospital, East Pilton Hospital, and Watts' Hospital, which is now a public school. He then sketched the present critical position of the voluntary hospitals, urged their maintenance on a voluntary basis by the organisation of "innumerable small sums" such as those which Hospital Saturday has long and successfully collected.

